# Supplementary material for: Diseases from the Spectrum of Dermatitis and Eczema: Can “Omics” Sciences Help with Better Systematics and More Accurate Differential Diagnosis?
Source: Int J Mol Sci. 2023 Jun 21;24(13):10468. doi: 10.3390/ijms241310468 (PMC10342122; doi:10.3390/ijms241310468)
Supplement: Supplementary file 1 [file ijms-24-10468-s001.zip › Spiewak_Dermatitis_IJMS_Supplementary_material.pdf]

Table S1. An ad-hoc PubMed query performed on the numbers of indexed research articles on “omics” research in the diseases from the spectrum of dermatitis and eczema.*

**Search for papers on…**	**PubMed query**	**All indexed**	**Last 5 years**
Omics in atopic dermatitis/eczema	atopic[title] AND (dermatitis[title] OR eczema[title]) AND (proteomics[title] OR genomics[title] OR metabolomics[title] OR transcriptomics[title] OR lipidomics[title] OR interactomics[title] OR proteomic[title] OR genomic[title] OR metabolomic[title] OR transcriptomic[title] OR lipidomic[title] OR interactomic[title] OR *omic*[title])	66	41
Omics in (any) dermatitis/eczema	(dermatitis[title] OR eczema[title]) AND (proteomics[title] OR genomics[title] OR metabolomics[title] OR transcriptomics[title] OR lipidomics[title] OR interactomics[title] OR proteomic[title] OR genomic[title] OR metabolomic[title] OR transcriptomic[title] OR lipidomic[title] OR interactomic[title] OR *omic*[title])	73	42
	**Percentage of papers devoted to atopic dermatitis:**	**90.4%**	**97.6%**

* performed on 28 February 2023. A simplistic assumption was undertaken that research papers devoted to application of omics in dermatitis/eczema with have either terms present in the title. No manual verification to exclude or include papers was undertaken. Despite the apparent overlapping, leaving any of the keywords off the query would result in missing some articles. Results restricted to human data.
